# Improvement of Heart Failure Discrimination by the Integration of the Left Ventricle Global Longitudinal Strain

**DOI:** 10.3390/clinpract16030055

**Published:** 2026-03-04

**Authors:** Alberto Cordero, Mª Amparo Quintanilla, Cristina Torres, Natalia López, Carles Bodí, Germán Bixquert, José Mª Lopez-Ayala

**Affiliations:** 1 Cardiology Department, Hospital Universitario de San Juan, 03550 San Juan de Alicante, Spain; mariamteati@hotmail.com (M.A.Q.); cristorresherves@gmail.com (C.T.); natlopez@gmail.com (N.L.); kharal1022@gmail.com (C.B.); german.bixvi@outlook.com (G.B.); josemaria_lopezayala@yahoo.es (J.M.L.-A.); 2 Grupo de Investigación Cardiovascular (GRINCAVA), Universidad Miguel Hernández, 03202 Elche, Spain; 3 Centro de Investigación Biomédica en Red de Enfermedades Cardiovasculares (CIBERCV), Av. Monforte de Lemos, 3-5, Pabellón 11, Planta 0, 28029 Madrid, Spain

**Keywords:** heart failure, GLS, echocardiography

## Abstract

**Introduction**: Clinical diagnosis of chronic heart failure (HF) in ambulatory patients can be difficult. Echocardiography is the most widespread diagnostic imaging technique, although the usefulness of the global longitudinal strain (GLS) of the left ventricle (LV) in this clinical setting is less clear. **Methods**: We performed a cross-sectional study of stable outpatients and GLS was obtained with an automatic software that uses the three apical planes of the LV. We analyzed the improvement of the diagnostic capacity of including GLS above all the clinical and echocardiographic parameters using reclassification indexes. **Results**: We included 1362 patients, including 12.9% with HF who presented lower values of ejection fraction (EF) and GLS and worse diastolic function. Most patients (92.8%) with HF had a GLS < −14 as compared to patients without HF (36.1%). LV EF (OR: 0.93) and GLS (OR: 1.27 CI 95% 1.20–1.35) were associated with the presence of HF. The AUC was significantly higher (*p* < 0.001) in the logistic model that included GLS vs. without GLS, and the reclassification index for GLS was 19.8%. GLS was more affected in patients with HFpEF vs. controls as well as diastolic function parameters. The logistic regression model only identified age (OR: 1.07 95% CI 1.02–1.06) and GLS (OR: 1.29 95% CI 1.21–1.38) as independently associated with the presence of HFpEF. The AUC of the model for the presence of HFpEF with GLS was significantly higher (*p* < 0.01). The reclassification index for GLS was 38.8%. **Conclusions**: LV GLS assessment increased the diagnostic discrimination of chronic HF in stable patients.

## 1. Introduction

Clinical diagnosis of clinically overt and decompensated heart failure (HF) is usually simple but the diagnosis of chronic HF can be much more challenging in the stable and outpatient clinics [1,2,3]. Shortness of breath is a common symptom of the patients referred to cardiology clinics where the diagnosis of HF should be clearly ruled out [4,5]. Echocardiogram is the most widely extended diagnostic imaging technique that provides reliable and reproductible information of left ventricle systolic and diastolic function [1,6]. Nearly 50% of the patients with HF have left ventricle ejection fraction (EF) > 0.50 and are classified as HF with preserved ejection fraction (HFpEF) although this diagnosis might be challenging [1].

Left ventricle global longitudinal strain (GLS) has been more recently validated as an effective measure for the evaluation of left ventricular function, especially in the absence of systolic dysfunction or in its earliest forms [7,8]. GLS is increasingly incorporated into routine echocardiographic assessment to enhance diagnostic precision and longitudinal patient management [8,9,10]. Even more, a recent meta-analysis identified that impaired GLS was significantly associated with adverse outcomes, with a hazard ratio of 1.36 (95% CI 1.11–1.67; *p* = 0.003) that clearly supports its determination [10].

HFpEF may carry prognostic information. However, the incremental diagnostic value of integrating GLS into routine clinical and echocardiographic evaluation in stable ambulatory patients has not been well quantified. Therefore, we tested whether adding GLS to standard clinical and echocardiographic parameters improves diagnostic discrimination for chronic HF and for HFpEF specifically, using model performance and reclassification metrics.

We analyzed whether routine assessment of GLS could improve the accuracy of chronic HF diagnosis in chronic stable patients.

## 2. Materials and Methods

We performed a retrospective study with all the consecutive patients remitted for an ambulatory echocardiogram in a single center between November 2023 and September 2024. Patients were classified by the presence or absence of a previous diagnosis of HF. The diagnosis of chronic HF had to be previously assigned by a certified physician report of any hospitalization with symptoms and signs of heart failure, an image test and elevated NT-pro BNP. To obtain a group without actual chronic HF we excluded patients with previous coronary heart disease, active or previous chemotherapy, pregnancy, thyroid diseases or systemic inflammatory diseases, such as lupus, systemic sclerosis, sarcoidosis or amyloidosis.

We collected 1730 echocardiograms. GLS could be not obtained in 135 (7.8%) patients. From 1595 patients with GLS available, we excluded 235 (15.9%), mainly for the presence of coronary heart disease (n = 180; 76.6%), valvular disease (n = 31, 13.2%), left bundle branch block (n = 12; 5.1%) or chemotherapy (n = 10; 4.3%). The final sample size was 1360 patients.

The antecedent of hypertension, diabetes mellitus or dyslipidemia was recorded by the presence in medical reports. Body mass index (BMI) was calculated as weight (kg)/height (m)^2^. Previous coronary heart disease was collected when there was an antecedent of myocardial infarction, acute coronary syndrome (with or without ST elevation), unstable angina or coronary revascularization.

Echocardiograms were performed with Phillips Epic EPIQ CVX. GLS was obtained with an automatic software that uses the 3 apical planes of the left ventricular (4, 2 and 3 chambers) as previously reported [8]. GLS was assessed using vendor-integrated two-dimensional speckle-tracking echocardiography (STE) software (Advanced Cardiac Motion Quantification, aCMQ; Philips Healthcare. Philips North America LLC. 222 Jacobs Street Cambridge, MA 02141, USA) on the Philips EPIQ CVx ultrasound system. Standard apical four-chamber, two-chamber, and long-axis (three-chamber) views were acquired during breath-hold with optimized frame rates (typically 40–90 frames/s). Endocardial borders were automatically detected and manually adjusted when necessary to ensure accurate tracking. The left ventricle was segmented according to the 17-segment American Heart Association (AHA) model. Longitudinal strain was calculated as the percentage change in myocardial length relative to baseline throughout the cardiac cycle, with negative values indicating myocardial shortening. Segmental peak systolic longitudinal strain values from all accepted segments were averaged to derive GLS. Analyses were performed on-cart, and only cardiac cycles with adequate tracking quality across all views were included. Impaired GLS was codified for values < −14 [8,10].

Relative wall thickness was calculated as 2*posterior wall thickness/end-diastolic diameter and values > 0.44 were considered as elevated [6].

The study obtained the exemption of the individual informed consent, according to current national regulations, for being a retrospective analysis. The study protocol was accepted by the Ethics in Investigation Committee of Hospital San Juan, Alicante in Spain (code 23/077 Tut).

The normality of the distribution for quantitative variables was assessed using the Shapiro–Wilk test. Because the quantitative variables followed a normal distribution, they were presented as means and standard deviations, and differences between groups were assessed using the Student *t*-test. Qualitative variables are described as absolute numbers (and percentages), and differences were tested via chi-squared tests. The variables associated with the presence of HF were assessed by logistic regression models. Variables that reached statistical significancy in the univariate analyses or could have a reliable implication were included in the models as covariates. The calibration of the model was tested by the Gronnesby and Borgan test and the calibration belt [10]. The diagnostic capacity of the models was assessed by the area under the curve (AUC) of the predicted probability of each model. For the assessment of the discriminative improvement of GLS we analyzed the difference in the AUC of the regression models without and with GLS as well as the net reclassification improvement (NRI) and the integrated discrimination improvement (IDI) [11].

## 3. Results

The clinical characteristics of the 1360 patients are presented in Table 1. The prevalence of chronic HF was 12.9% (n = 176). As expected, patients with chronic HF had lower EF and GLS values but statistically significant differences were observed in all clinical characteristics. Most patients (92.8%) with HF had a GLS < −14 as compared to patients without HF (36.1%).

**Figure 1:**
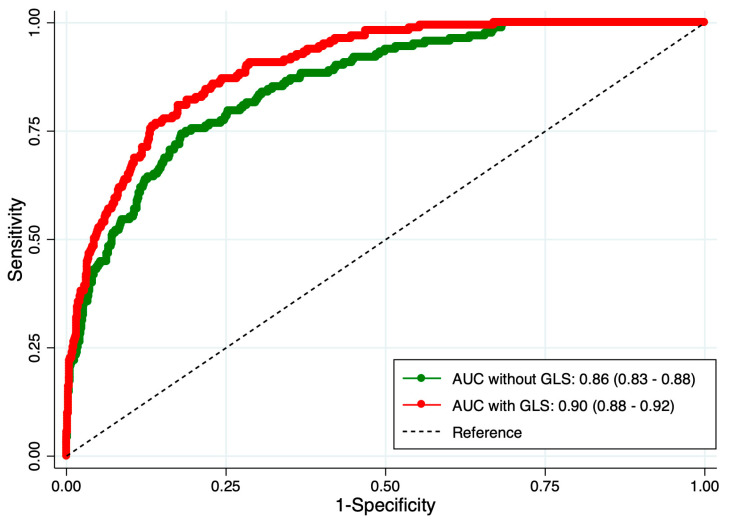
Receiver operation curve for the regression model without and with the global longitudinal strain (GLS) for the presence of heart failure.

The logistic regression model, adjusted for age, sex, hypertension, diabetes and left ventricle end-diastolic diameter, identified an association of left ventricle EF (OR: 0.93 CI 95% 0.91–0.95) and GLS (OR: 1.27 CI 95% 1.20–1.35) with the presence of HF. As shown in Figure 1, the AUC was significantly higher (*p* < 0.001) in the logistic model that included GLS vs. without GLS. The calibration and accuracy of the models were high (Supplementary Figure S1). The reclassification index for GLS was 19.8% (14.1–25.0), the INRI was 1.06 (0.86–1.23), and the IDI 0.13 (0.09–1.19).

We also assessed the differences among patients with HFpEF and HF with reduced left ventricle EF. As shown in Table 2, patients with HFpEF had higher BMI, female gender and lower prevalence of coronary heart disease; in the echocardiographic parameters, they also exhibited shorter end-diastolic diameter but higher relative wall thickness. GLS was slightly better in patients with HFpEF although the prevalence of GLS > −14 was similar in both types of HF; surprisingly, no differences were observed in diastolic parameters.

We finally investigated the differences between patients with HFpEF compared to controls without chronic HF. As shown in Table 3, patients with HFpEF had higher mean age, higher BMI, higher prevalence of hypertension, diabetes and atrial fibrillation. According to echocardiogram findings, patients with HFpEF had larger left ventricle end-diastolic diameter, indexed volume and mass and left atrial volume; nonetheless, no differences were observed in RWT. GLS was more affected in patients with GLS (−9.5 (3.2) vs. −14.7 (4.4); *p* < 0.001) as well as diastolic function parameters.

**Figure 2:**
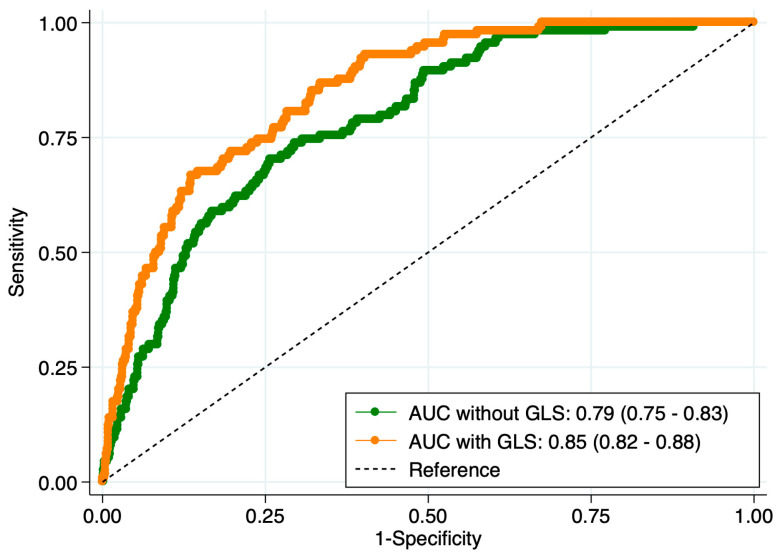
Receiver operation curve for the regression model without and with the global longitudinal strain (GLS) for the presence of heart failure with preserved ejection fraction.

The logistic regression model identified age (OR: 1.07 95% CI 1.02–1.06) and GLS (OR: 1.29 95% CI 1.21–1.38) as independently associated with the presence of HFpEF. The calibration and accuracy of the models were fair (Supplementary Figure S2). We finally assessed the diagnostic performance of the model without and with GLS and, as shown in Figure 2, the AUC of the model with GLS was significantly higher (*p* < 0.01). The reclassification index for GLS was 38.8% (14.1–25.0), the INRI was 0.96 (0.80–1.11), and the IDI 0.12 (0.08–1.16).

## 4. Discussion

The main results of our study are that most patients with HF have impaired GLS, regardless of the left ventricular EF, and its assessment improves the discriminative improvement of HF. Similarly, age and GLS were the only two variables independently associated with the presence of HFpEF. The rate of reclassification with GLS was much higher for the presence of HFpEF vs. controls, underscoring the potential of GLS as a transformative diagnostic tool in HFpEF [7,8,9]. The definitive discrimination of heart failure (HF) in ambulatory patients can be challenging, and our results suggest that routinely evaluating GLS may be useful for the final diagnosis. The key novelty of our study is not simply confirming that GLS is frequently impaired in HF and HFpEF, but demonstrating that adding GLS to routine clinical and echocardiographic assessment provides measurable incremental discrimination value in a real-world ambulatory setting. In a large cohort of consecutive stable outpatients, GLS significantly improved model discrimination (AUC) and yielded clinically meaningful net reclassification, particularly for HFpEF (NRI 38.8%), indicating that a substantial proportion of patients would be more accurately classified when GLS is incorporated into the diagnostic framework.

Traditional reliance on left ventricular EF often fails to detect subtle myocardial dysfunction in some patients, leading to diagnostic delays and suboptimal management. GLS provides a more sensitive and nuanced assessment of myocardial function, enabling earlier and more accurate discrimination of HF [7,8,11]; this might be particularly valuable for distinguishing HFpEF from other conditions that mimic its symptoms, such as pulmonary hypertension or obesity-related dyspnea [12]. The rate of reclassification for HFpEF was 38%, reflecting that more than one third of the patients would be precisely discriminated by the inclusion of GLS. Recent reports have highlighted that conventional echocardiographic determinations have a low diagnostic performance of HFpEF [13]. This study quantifies the incremental diagnostic and reclassification benefit of routine GLS integration for identifying chronic HF and HFpEF in stable outpatients, rather than only describing GLS impairment. No clinical trial has been conducted to assess GLS changes with medical therapies, but our results could help to understand the prevention of HF with some therapies, such as SGLT2 inhibitors [14], in patients without a previous diagnosis of HF because such diagnosis was based only on left ventricle EF.

GLS is recommended as a sensitive addition to left ventricular EF for detecting subclinical systolic dysfunction and for refining risk stratification across a broad range of cardiovascular diseases [9]. Current guidelines support its routine use in cardio-oncology surveillance, where a relative reduction of at least 15% from baseline is considered clinically significant [15]. The incorporation of GLS into routine clinical workflows is feasible [8], as most modern echocardiography systems already support its analysis. GLS is a continuous variable and there is a lack of a clear cut-off value for normality; we selected GLS-14, as previously reported [7,8], and most patients with HFpEF had an impaired GLS, suggesting that GLS could better characterize the left ventricular function in this clinical setting. Results are also in concordance with the reclassification and the discriminative improvement after inclusion of GLS in the regression models. We excluded patients with chronic coronary syndrome since they have higher risk of HF, even if left ventricular EF is >0.50 [16]. Diastolic parameters were similar in patients with HFpEF compared to patients with HF and reduced ejection fraction, and differences were mainly observed in left ventricular EF, GLS and left ventricular dimensions. HFpEF is a very heterogeneous syndrome [1], not fully understood, and our results support the role of left ventricular mechanics beyond diastolic function.

Our results might be limited by the fact that this was a retrospective and single center study performed only in stable patients. In order to minimize the selection bias, we include all consecutive patients. The diagnosis of HF was not adjudicated or deeply reviewed since it was registered if it was documented in the medical reports. Similarly, the echocardiograms were performed and analyzed by the same operator and no inter-observer comparisons could be analyzed. The exclusion criteria were defined to minimize the confusion and interactions of other conditions although it might, as well, affect the external validation in clinical practice where many other concomitant comorbidities are usually present. This study is based on daily practice and no intra-observer variability could be assessed as echocardiograms are not usually reviewed by two operators. Reproducibility of GLS measurements was not formally assessed in this study, and intra- and inter-observer variability data are therefore not available. As GLS represents a central variable in our analysis, this constitutes an important methodological limitation. However, strain analysis was performed using vendor-integrated semi-automated software from three standard apical views, which reduces operator dependency compared with fully manual approaches. Furthermore, examinations were performed in a high-volume center by experienced operators, reflecting routine clinical practice. Published data suggest that modern vendor-based GLS measurements generally demonstrate good reproducibility, with relatively low intra- and inter-observer variability. Given the magnitude of the observed differences in GLS and the significant improvement in model discrimination and reclassification after GLS inclusion, it is unlikely that measurement variability alone explains our findings. Finally, some associations might be limited by confounding variables that were not collected and, therefore, could not be taken under consideration. Nonetheless, since clinical features of our population are similar to previous reports [7,8,16,17,18,19] we believe that our results might be representative and clinically meaningful.

## 5. Conclusions

Left ventricular GLS assessment increases the discriminative ability of clinical and echocardiographic variables for chronic HF patients. The inclusion of GLS is particularly transformative for the discrimination HFpEF, where it was identified alongside age as an independent predictor and yielded a high reclassification index of 38.8%. This clinical value is reinforced by the significant improvement in discriminative accuracy, as reflected by higher AUC. GLS provides a more sensitive and nuanced assessment of left ventricle mechanics than the echocardiographic measurement alone. Furthermore, the feasibility of using automatic software makes the routine assessment of GLS an efficient and accessible tool for optimizing the management of stable ambulatory patients in daily clinical practice.

## Figures and Tables

**Table 1:** Clinical and echography of the population according the diagnosis of heart failure.

	Total	Heart Failure		*p*
		No	Yes	
N	1360	1184 (87.1%)	176 (12.9%)	
Age	59.3 (16.9)	57.2 (17.0)	71.2 (10.7)	<0.01
BMI (kg/m^2^)	27.7 (5.5)	27.5 (5.4)	29.3 (5.4)	<0.01
Women (%)	43.9	45.6	34.2	<0.01
Diabetes (%)	16.8	11.6	46.3	<0.01
Hypertension (%)	48.8	44.6	73.1	<0.01
Dyslipidemia (%)	16.6	14.8	26.9	<0.01
Current smokers (%)	5.8	6.3	2.5	0.01
Atrial fibrillation (%)	14.4	10.6	36.3	<0.01
End-diastolic diameter (mm)	49.8 (9.5)	48.5 (8.7)	56.8 (10.8)	<0.01
Indexed end-diastolic volume (mL/m^2^)	59.9 (21.4)	56.6 (14.4)	79.1 (38.5)	<0.01
Indexed mass (g/m^2^)	101.3 (35.2)	96.8 (30.3)	127.5 (48.0)	<0.01
RWT	0.43 (0.1)	0.43 (0.1)	0.39 (0.1)	<0.01
RWT > 0.44 (%)	41.4%	43.39%	29.8%	<0.01
Left atrial volume (mL)	50.6 (43.5)	47.3 (44.1)	69.6 (33.5)	<0.01
LVEF (M-mode)	60.7 (10.2)	62.5 (8.2)	50.7 (13.8)	<0.01
LVEF (Simpson)	57.3 (9.7)	59.1 (7.6)	47.5 (14.0)	<0.01
GLS	−14.3 (4.8)	−15.2 (4.4)	−8.9 (3.2)	<0.01
E/É ratio	8.3 (4.3)	7.9 (3.8)	11.1 (5.7)	<0.01
E/É ratio > 14 (%)	7.9	5.6	21.0	<0.01
TAPSE	26.9 (11.7)	27.4 (12.4)	23.6 (5.8)	<0.01

BMI: body mass index; GLS: global longitudinal strain; LVEF: left ventricle ejection fraction; RWT: relative wall thickness; TAPSE: tricuspid annular plan systolic excursion.

**Table 2:** Clinical and echography characteristics of only patients with heart failure with preserved vs. reduced ejection fraction.

	Heart Failure		*p*
	EF ≤ 50	EF > 0.50	
N	49 (27.8%)	127 (72.2%)	
Age	69.0 (10.5)	72.4 (10.5)	0.06
BMI (kg/m^2^)	28.0 (5.0)	30.0 (5.7)	0.03
Women (%)	20.4	40.9	0.01
Diabetes (%)	55.10	42.9	0.14
Hypertension (%)	63.3	77.0	0.07
Dyslipidemia (%)	26.5	27.8	0.87
Current smokers (%)	4.1	1.6	0.25
Previous CHD (%)	26.5	9.5	0.01
Atrial fibrillation (%)	26.5	40.5	0.09
End-diastolic diameter (mm)	63.4 (12.1)	53.5 (8.9)	<0.01
Indexed end-diastolic volume (mL/m^2^)	112.3 (47.1)	64.1 (23.1)	<0.01
Indexed mass (g/m^2^)	142.7 (65.5)	122.6 (38.6)	0.01
RWT	0.3 (0.1)	0.4 (0.1)	<0.01
RWT > 0.44 (%)	14.3	39.8	<0.01
Left atrial volume (mL)	69.1 (34.9)	68.6 (31.4)	0.92
LVEF (M-mode)	37.2 (12.5)	56.7 (10.1)	<0.01
LVEF (Simpson)	30.7 (8.3)	55.2 (7.8)	<0.01
GLS	−7.6 (3.2)	−9.5 (3.2)	0.01
GLS > −14 (%)	98.0	90.7	0.91
E/É ratio	11.8 (4.8)	10.6 (5.8)	0.20
E/É ratio > 14 (%)	28.6	18.3	0.14
TAPSE	22.7 (6.4)	24.3 (5.5)	0.12

BMI: body mass index; CHD: coronary heart disease; GLS: global longitudinal strain; LVEF: left ventricle ejection fraction; RWT: relative wall thickness; TAPSE: tricuspid annular plan systolic excursion.

**Table 3:** Clinical and echography characteristics of patients with without heart failure vs. patients with heart failure with preserved reduced ejection fraction.

	Total	Heart Failure		*p*
	Yes	No	HFpEF	
N	1311	1184 (90.3%)	127 (9.7%)	
Age	60.5 (16.7)	59.5 (16.7)	72.4 (10.5)	<0.01
BMI (kg/m^2^)	27.8 (5.3)	27.7 (5.3)	30.0 (5.7)	0.03
Women (%)	41.9	42.0	40.9	0.81
Diabetes (%)	17.8	17.8	42.9	<0.01
Hypertension (%)	49.8	47.5	77.0	<0.01
Dyslipidemia (%)	21.2	20.7	27.8	0.06
Current smokers (%)	6.1	6.5	1.6	0.01
Atrial fibrillation (%)	12.4	10.1	40.5	<0.01
End-diastolic diameter (mm)	49.3 (8.4)	49.0 (8.3)	53.5 (8.9)	<0.01
Indexed end-diastolic volume (mL/m^2^)	58.3 (15.8)	57.8 (15.0)	64.1 (23.1)	<0.01
Indexed mass (g/m^2^)	103.8 (91.6)	102.3 (94.5)	122.6 (38.6)	0.01
RWT	0.4 (0.1)	0.4 (0.1)	0.4 (0.1)	0.66
RWT > 0.44 (%)	42.9	43.1	39.8	0.48
Left atrial volume (mL)	50.8 (48.6)	49.3 (49.4)	68.6 (31.4)	>0.01
LVEF (M-mode)	58.1 (8.2)	58.4 (8.2)	56.7 (10.1)	<0.01
LVEF (Simpson)	58.1 (8.2)	58.4 (8.2)	55.2 (7.8)	<0.01
GLS	−14.3 (4.6)	−14.7 (4.4)	−9.5 (3.2)	0.01
GLS > −14 (%)	45.6	41.9	90.7	<0.01
E/É ratio	8.7 (6.3)	8.6 (6.3)	10.6 (5.8)	<0.01
E/É ratio > 14 (%)	8.3	7.5	18.3	<0.01
TAPSE	26.8 (10.8)	27.0 (11.1)	24.3 (5.5)	0.12

BMI: body mass index; GLS: global longitudinal strain; LVEF: left ventricle ejection fraction; RWT: relative wall thickness; TAPSE: tricuspid annular plan systolic excursion.

## Data Availability

Data could be provided under a reasonable proposal.

## Supplementary Materials

The following supporting information can be downloaded at: https://www.mdpi.com/article/10.3390/clinpract16030055/s1, Figure S1: Calibration belt for the assessment of the calibration and accuracy of the logistic regression model assessing the presence of heart failure; Figure S2: Calibration belt for the assessment of the calibration and accuracy of the logistic regression model assessing the presence of heart failure with preserved ejection fraction.

## Abbreviations

The following abbreviations are used in this manuscript:

HF	Heart failure
HFpEF	Heart failure with preserved ejection fraction
GLS	Global longitudinal strain
