# Supplementary figures and images for: Improvement of Heart Failure Discrimination by the Integration of the Left Ventricle Global Longitudinal Strain
Source: Clin Pract. 2026 Mar 4;16(3):55. doi: 10.3390/clinpract16030055 (PMC13025956; doi:10.3390/clinpract16030055)

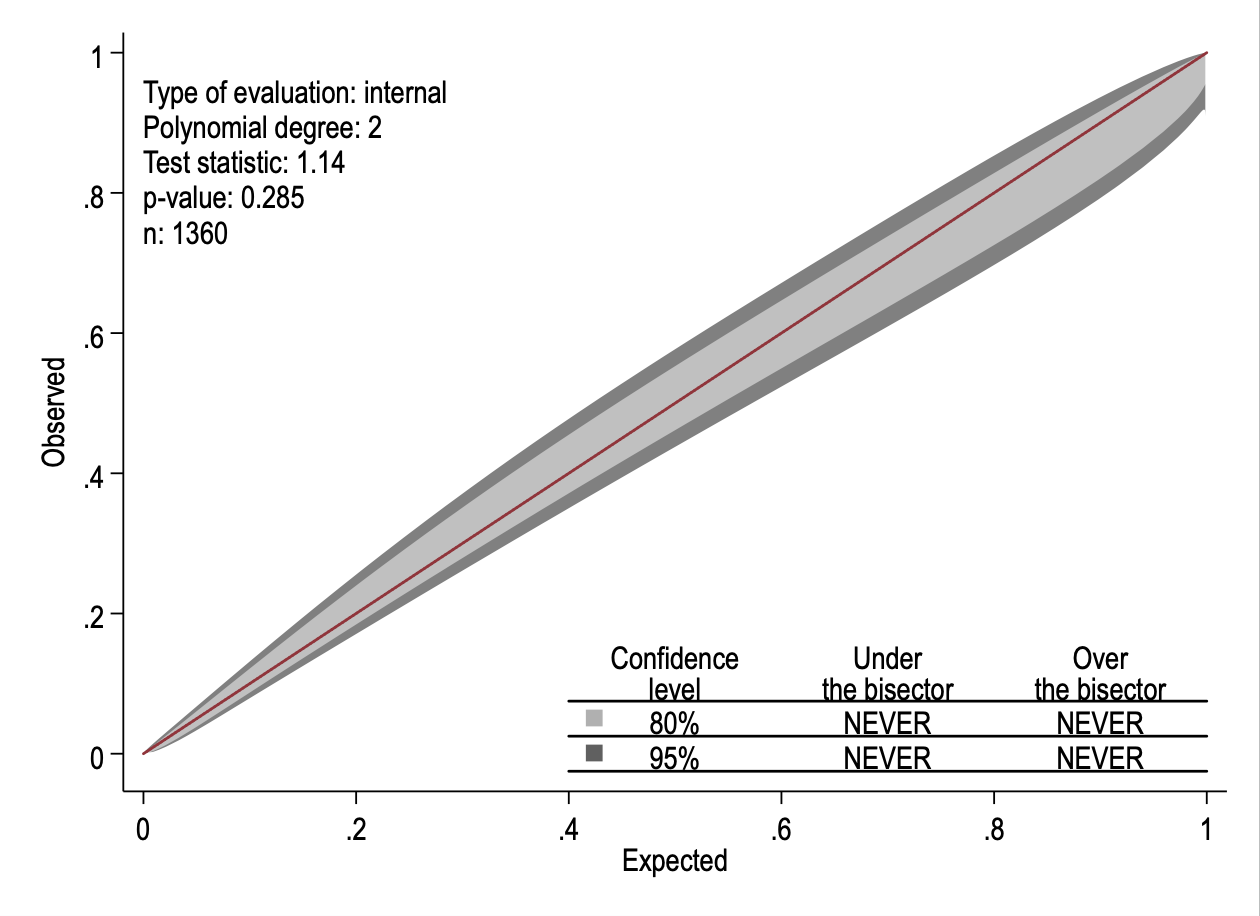

Supplement: Supplementary file 1 [file clinpract-16-00055-s001.zip › suppl Figure S1.tif]

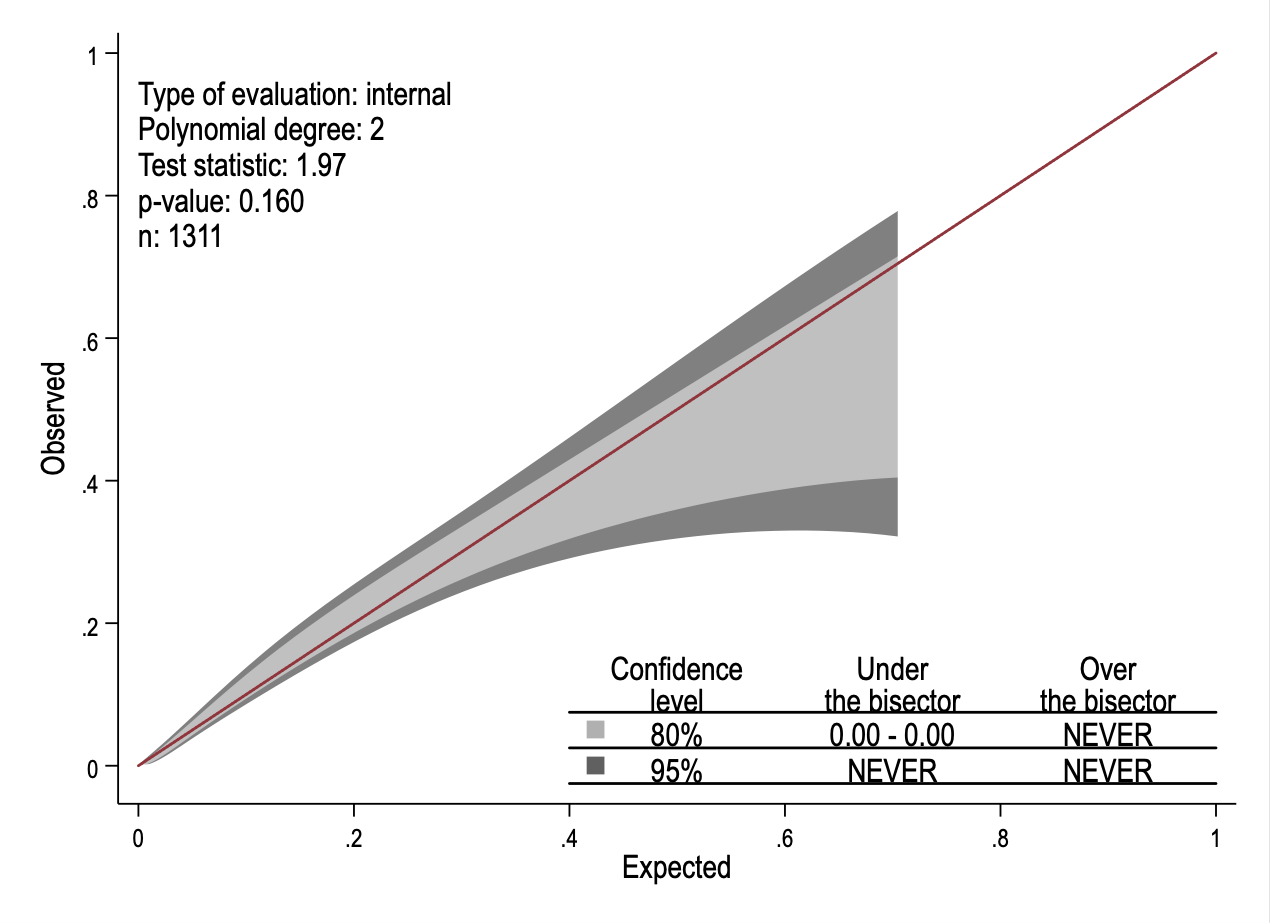

Supplement: Supplementary file 1 [file clinpract-16-00055-s001.zip › suppl Figure S2.tif]
